# COVID‐19 and ophthalmology: An environmental work hazard

**DOI:** 10.1002/1348-9585.12124

**Published:** 2020-07-21

**Authors:** Irene C. Kuo, Terrence P. O’Brien

**Affiliations:** ^1^ Wilmer Eye Institute Johns Hopkins University School of Medicine Baltimore MD USA; ^2^ Bascom Palmer Eye Institute University of Miami Miller School of Medicine Miami FL USA

**Keywords:** 2019‐nCoV, conjunctivitis, coronavirus, COVID‐19, occupational health, ophthalmology, SARS‐CoV‐2

Studies from large clinical series indicate that conjunctival congestion or conjunctivitis can be a clinical manifestation of coronavirus 19 (COVID‐19),^1^, ^2^ with a 0.8% prevalence of the former in 1099 confirmed cases in China.^1^ In confirmed patients having conjunctivitis, SARS‐CoV‐2 has been recovered from conjunctival secretions, posing a potential route of ocular transmission.^2^ Although generally considered a surgical subspecialty, ophthalmology involves medical care with close contact with patients’ aerosolized respiratory droplets or tears and often manipulation of eyelids and conjunctiva during biomicroscopic slit lamp examination. Few (if any) ophthalmologists routinely wear respiratory masks or gloves unless the patient appears to have a respiratory infection or conjunctivitis with tearing or other discharge. It is not widely known that the late Dr Li Wenliang, who alerted Chinese government officials of an incipient viral syndrome that would be named COVID‐19, was a young ophthalmologist. By his account, he believed he contracted the infection from a glaucoma patient he was evaluating for conjunctivitis; a month after exposure, he succumbed to respiratory failure. With the outbreak of COVID‐19, caused by severe acute respiratory syndrome coronavirus 2 (SARS‐CoV‐2) (formerly “2019‐nCoV”), is a growing realization that ophthalmologists may be the first to identify patients with COVID‐19 before they manifest respiratory symptoms. Thus, ophthalmologists may be inherently at increased occupational risk for infection. The essential nature of encounters in ophthalmological clinics necessitates patient and ophthalmologist to be separated by less than 20 cm during slit‐lamp biomicroscopic examination, and less than 5 cm in situations requiring direct ophthalmoscopy, yet the World Health Organization recommends that one “maintain at least 1 meter (3 feet) distance between yourself and anyone who is coughing or sneezing”; the Centers for Disease Control and Prevention recommends maintaining a distance of 6 feet from anyone. “Social distancing” is not an option for practicing ophthalmologists.

Cataracts and macular degeneration, two common ophthalmic conditions, are associated with aging. Advanced age is a known risk factor for susceptibility to COVID‐19. Thus, additional increased risk portended to ophthalmic practice relates to the increased age of patients in ophthalmology clinics or undergoing ophthalmic procedures.

Patients with acute conjunctivitis present to a variety of providers, from ophthalmologists to internists to family care physicians to emergency room providers. However, only ophthalmologists assume an intimacy that routinely utilizes the slit‐lamp biomicroscope for closely examining patients within a proximate nominal hazard zone. As physicians, ophthalmologists also understand that many systemic viral infections have ocular manifestations; examples include adenovirus,^3^ enterovirus 70, H1N1, SARS coronavirus (SARS‐CoV), and human coronavirus NL63 (HCoV‐NL63).^4^ HCoV‐NL63 was, in fact, first identified in the Netherlands in a 7‐month‐old child with conjunctivitis and bronchiolitis.^4^ Therefore, it may not be surprising that ophthalmologist Dr Li was able to deduce an incipient SARS‐like viral outbreak in Wuhan, China, well in advance of others as coronavirus syndromes are known to be associated with conjunctivitis.

In a 2004 series of 36 patients with suspected SARS‐CoV,^5^ the three with SARS‐CoV RNA in their tears had sampling performed early in the course of their illness (within 9 days of symptom onset); whether these patients had conjunctivitis was not specified. The authors hypothesized that viral secretion in tears occurs only during the early phase of SARS‐CoV. SARS‐CoV‐2 has also been found in tears of one infected patient by reverse‐transcription polymerase chain reaction (RT‐PCR).^2^ In a recent study of 30 patients in China diagnosed with COVID‐19 based on RT‐PCR of blood or sputum and radiographic findings consistent with viral pneumonia, cases were classified as “common” or “severe” based on respiratory involvement or organ failure. This study showed that SARS‐CoV‐2 existed in the tears and conjunctival secretions of the one patient with conjunctivitis (and “common” disease), but not in tears or secretions of the 29 patients without conjunctivitis.

Ophthalmologists have long been aware that tears/ocular secretions can contain transmissible, highly contagious pathogens (eg human immunodeficiency virus, adenovirus, and hepatitis C). Patients who present to ophthalmologists with isolated conjunctivitis may already be infected and secreting SARS‐CoV‐2 in tears and elsewhere. Thus, universal precautions that should be programmed into standard ophthalmic practice apply more than ever. One study has raised the possibility of different viral shedding routes and questioned whether evidence of positive RT‐RNA in respiratory specimens is sufficient for diagnosis. It appears that patients can be viremic or have positive anal swabs later in the disease course but have negative respiratory specimens by that time.^6^Patients with two such negative swabs would be considered SARS‐CoV‐2‐negative and released, yet possibly they could transmit disease through alternate routes.

Although it appears no one studied whether SARS‐CoV was present in convalescent patients or whether tears were infectious, ophthalmologists should probably don protective equipment when examining certain groups of patients in the pandemic caused by SARS‐CoV‐2. One group would be patients with signs and symptoms of upper respiratory infection such as fever and shortness of breath, regardless of conjunctival findings. The other group would be patients with conjunctivitis and possessing risk factor(s) (travel to high‐risk areas or contact with persons who have returned from such areas or persons with known infection). With the SARS outbreak in 2004, Singaporean ophthalmologists wrote, “Stringent barrier methods using the ‘M3G’ (mask, gown, gloves, and goggles) should be the gold standard when dealing with suspected SARS patients.”^6^ These recommendations make sense for ophthalmologists in 2020 when examining high‐risk patients. Gloves should be worn and discarded after each patient encounter. Hand hygiene is important; ophthalmologists should use an alcohol‐based hand sanitizer or wash hands with soap and water for 30 seconds before and after every patient encounter. Sterile cotton tip applicators can be used to manipulate eyelid and ocular tissues.

It is better to avoid exposure than try to contain exposures. At the front end of protection of patients and ophthalmology personnel is instituting screening measures prior to patient arrival. Hong Kong provides one example. Starting January 25, 2020, the Emergency Response Level was activated in all public hospitals, and the ophthalmology departments of two hospitals serving the Kowloon peninsula with a catchment area of 1.1 million people adopted even stricter standards to curb disease transmission in ophthalmology patients.^7^ These measures included reduction in clinic volume and number of elective procedures by short message service (ie texts) to patients to postpone non‐urgent appointments; requirement of all ophthalmology personnel to wear face masks; screening at the clinic entrance of all ophthalmology patients and accompanying persons by infrared thermometers; administering questionnaires to afebrile patients inquiring of occupation and travel to affected areas or contact with person(s) traveling to such areas or contact with a suspected or confirmed case within 14 days. Symptoms of upper respiratory tract infection and acute conjunctivitis were also used to screen patients. If any of the above were affirmative, the patient's appointment was postponed for at least 14 days. Ophthalmologists performing dacryocystorhinostomy must don personal protective equipment when performing nasal endoscopy; general anesthesia cases were re‐evaluated given the risk of generating aerosols from endotracheal intubation.^7^

As ophthalmologists may be the first medical contact a newly infected patient makes in seeking care for acute conjunctivitis, they have an essential role in guiding infection control measures to prevent unnecessary exposures to other patients, staff, doctors, and the community. Like Dr Li, ophthalmologists may be the first to diagnose a more serious condition than isolated conjunctivitis. And like Dr Li, without the measures described above, ophthalmologists may face a potentially lethal occupational hazard.

## AUTHOR CONTRIBUTIONS

TPOB is responsible for conception and design of submitted work; acquisition, analysis, and interpretation of data; drafting and revising the work for intellectual content; final approval of submitted version; and agreement to be accountable for all aspects of the work regarding the accuracy and integrity of the work.
